# Effect of a guide for clinical reasoning on Nursing students’ diagnostic accuracy: A clinical trial

**DOI:** 10.1590/1518-8345.5452.3515

**Published:** 2022-03-21

**Authors:** Aline Batista Maurício, Elaine Drehmer de Almeida Cruz, Alba Lucia Bottura Leite de Barros, Mary Gay Tesoro, Camila Takao Lopes, Anne Marie Simmons, Juliana de Lima Lopes, Lidia Santiago Guandalini

**Affiliations:** 1 Universidade Federal do Paraná, Curitiba, PR, Brasil.; 2 Bolsista da Coordenação de Aperfeiçoamento de Pessoal de Nível Superior (CAPES), Brasil.; 3 Universidade Federal de São Paulo, Escola Paulista de Enfermagem, São Paulo, SP, Brasil.; 4 City University of New York, Lehman College, Nova York, NY, Estados Unidos da América.; 5 City University of New York, School of Professional Studies, Nova York, NY, Estados Unidos da América.

**Keywords:** Critical Thinking, Clinical Decision-Making, Nursing Students, Randomized Controlled Trial, Teaching, Nursing, Pensamento Crítico, Tomada de Decisão Clínica, Estudantes de Enfermagem, Ensaio Clínico Controlado Randomizado, Educação em Enfermagem, Enfermagem, Pensamiento, Toma de Decisiones Clínicas, Estudiantes de Enfermería, Ensayo Clínico Controlado Aleatorio, Educación en Enfermería, Enfermería.

## Abstract

**Objective:**

to evaluate the effect of the Self-Instructional Guide for Clinical Reasoning on the diagnostic accuracy of undergraduate Nursing students.

**Method:**

a randomized, parallel and double-blind (researchers and outcome evaluators) clinical trial, carried out with undergraduate Nursing students. Validated case studies were applied in two phases to identify the patient’s Nursing diagnosis/problem, etiology and clues, using the Guide with the intervention group in the second phase. The outcomes - diagnostic and etiological accuracy and number of clues - were evaluated using validated rubrics. Descriptive statistics were used to analyze demographic data; Fisher’s exact test for similarities in prior education and confidence; Mann-Whitney’s test for age; and non-parametric ANOVA test in the evaluation of the hypothesis of differences in performance.

**Results:**

final sample composed of 24 students in the control group and 27 in the intervention group; no difference as to gender, age and schooling. There was a difference in diagnostic (p=0.041) and etiological (p=0.0351) accuracy in the intervention group, showing a negative effect of using the Guide.

**Conclusion:**

the one-time self-instruction was not effective in impacting the diagnostic accuracy of students solving case studies. Repeated application of the Guide as a teaching tool can be effective in improving such outcome. REBEC: RBR-4bhr78.

## Resumo

**Objetivo:**

avaliar o efeito do Guia Autoinstrucional para Raciocínio Clínico na acurácia diagnóstica de estudantes de Bacharelado em Enfermagem.

**Método:**

ensaio clínico randomizado, paralelo, duplo cego (pesquisadores e avaliadores dos desfechos), realizado com graduandos de Enfermagem. Aplicaram-se, em duas fases, estudos de caso validados para a identificação de diagnóstico de enfermagem/problema do paciente, etiologia e pistas, sendo utilizado o Guia com o grupo intervenção na segunda fase. Os desfechos - acurácia diagnóstica, etiológica e número de pistas - foram avaliados por meio de rubricas validadas. Para análise, utilizou-se estatística descritiva para dados demográficos; teste exato de Fisher para similaridades na educação prévia e confiança; teste de Mann-Whitney para idade; teste ANOVA não paramétrico na avaliação da hipótese de diferenças no desempenho.

**Resultados:**

amostra final composta por 24 estudantes no grupo controle e 27 no intervenção; sem diferença quanto ao sexo, idade e educação. Houve diferença para acurácia diagnóstica (p=0,041) e etiológica (p=0,0351) no grupo intervenção, mostrando efeito negativo da utilização do Guia.

**Conclusão:**

a autoinstrução implementada uma única vez não foi efetiva em impactar a acurácia diagnóstica de estudantes solucionando estudos de caso. A aplicação repetida do Guia como ferramenta didática pode ser efetiva para melhorar tal desfecho. REBEC: RBR-4bhr78.

## Resumen

**Objetivo:**

evaluar el efecto de la Guía Autoinstruccional de Razonamiento Clínico en la precisión diagnóstica de estudiantes de Licenciatura en Enfermería.

**Método:**

ensayo clínico aleatorizado, paralelo, doble ciego (investigadores y evaluadores de resultados), realizado con estudiantes de la carrera de enfermería. Se aplicaron estudios de casos validados en dos fases para identificar el diagnóstico de enfermería/problema del paciente, etiología y pistas, utilizando la Guía con el grupo experimental en la segunda fase. Los resultados (precisión diagnóstica, etiológica y número de pistas) se evaluaron utilizando rúbricas validadas. Para el análisis se utilizó estadística descriptiva para datos demográficos; prueba exacta de Fisher para similitudes en educación previa y confianza; prueba de Mann-Whitney para la edad; prueba ANOVA no paramétrica en la evaluación de la hipótesis de diferencias en el desempeño.

**Resultados:**

muestra final compuesta por 24 estudiantes en el grupo control y 27 en el grupo experimental; no había diferencias en cuanto al sexo, la edad y educación. Hubo diferencia en la precisión diagnóstica (p=0,041) y etiológica (p=0,0351) en el grupo experimental, que mostraron un efecto negativo del uso de la Guía.

**Conclusión:**

la autoinstrucción implementada por única vez no fue efectiva para generar un impacto en la precisión diagnóstica de los estudiantes que resolvieron los estudios de casos. La aplicación repetida de la Guía como herramienta de enseñanza puede ser eficaz para mejorar dicho resultado. REBEC: RBR-4bhr78.

**Highlights:**

(1) A guide with the potential to associate active methodologies, even in virtual environments.

(2) It can favor the students’ autonomy and place them as central actors in learning.

(3) It contributes to the identification of the priority Nursing Diagnoses.

(4) It engages the students to undertake challenging activities.

(5) It highlights important reasoning points to favor patient safety.

## Introduction

Clinical reasoning is an essential competence for nurses’ professional practice. It is considered crucial that its development begins during training^1^ ^-^ ^3^. Facilitating the development of reasoning is a challenge for the educators due to its complexity and multifaceted nature; using a strategy in which the student actively participates is a way to facilitate this process^4^. 

Among the facilitators of the development of clinical reasoning, the use of the Nursing Process (NP) can be pointed out, as it contributes to the organization of thought in caring for people. The NP is considered a model of critical thinking, conducive to the promotion of quality care, given the scope of the actions carried out, and the necessary basis for decision-making^1^. 

The NP consists of five interrelated phases: data collection, Nursing Diagnosis (ND), planning, implementation and evaluation, with the recommendation that the Nursing team implements them in all environments where Nursing care is provided^5^. The diagnostic stage corresponds to data analysis and interpretation and represents a “[...] clinical judgment about a human response to health conditions/life processes, or susceptibility to such a response, of an individual, a family, a group or a community”^6^. Identifying precise NDs directs the choice of assertive interventions to improve the patient’s outcomes. On the other hand, selection of inaccurate NDs can lead to the implementation of unnecessary interventions and neglect of human responses that are a priority, which incurs in the possibility of causing adverse events, increased hospitalization times and higher financial costs for the institution^7^ ^-^ ^9^. 

Such being the case, the development of clinical reasoning is encouraged, aiming to promote better performance in indispensable skills, decision-making, quality and safety when assisting the person^2^ ^-^ ^3^. A number of studies point to gaps in the use of strategies that promote the construction of the Nursing student’s knowledge and autonomy and that also assist in the development of critical thinking and clinical reasoning^4^, problem-solving and clinical decision-making skills, which are incorporated into the curricula of Nursing Schools and Colleges, as well as the use of instruments to assess these phenomena^10^. This fact was evident in the coronavirus (COVID-19) pandemic, during which the educational institutions acknowledged the need for changes in care, management and research, as well as in health education, raising awareness of the need to incorporate teaching and learning to the reality of the students’ living conditions^11^. Although there are some studies on teaching strategies in various modalities, there is scarcity of research studies with higher levels of evidence, as well as of studies that include reflective and innovative strategies for the improvement of clinical reasoning^(2.4)^. In addition to that, the need for strategies to be tested and for new instruments to be made available is made explicit, in order to accurately assess teaching-learning strategies^4^. 

In this sense, an American woman researcher developed a Written Clinical Reasoning Prompt (WCRP) - in Brazil, named “Self-Instructional Guide to Clinical Reasoning referred to as “Introduction on how to analyze a case: Think like a nurse”, based on the Developing Nurses’ Thinking (DNT) model^12^, a tool used to guide the students during the resolution of clinical case studies. This guide has 11 sentences, distributed into four sections, which direct the student to identify and analyze diverse evidence relevant to a case, as well as to select the patient’s priority diagnosis or problem, with considerations about their safety^13^. The instrument was adapted and translated into Brazilian Portuguese, with satisfactory evidence of content and face validity in one of the stages of the multicenter study^14^. 

Previously, WCRP was pilot-tested with 11 Nursing students from the last period of the undergraduate course at an American university, for assessment regarding language, readability and perception in terms of helping to facilitate the clinical reasoning process. The case studies were submitted to content validation by six expert nurses and by the same 11 Nursing students from the last semester of the aforementioned university^13^.

Given the relevance of developing clinical reasoning for Nursing, this research aimed at evaluating the effect of WCRP on the diagnostic accuracy of undergraduate Nursing students during the resolution of clinical case studies. The hypotheses tested were that accuracy in identifying the ND, etiologies and number of clues would be increased in Nursing students who use WCRP to solve a case study, when compared to those who do not. The development of the body of knowledge on clinical reasoning and diagnostic accuracy in Nursing in professional training stands out as relevant, aiming at care quality and patient safety^13^. 

## Method

### Study type

This is a randomized, parallel and double-blind clinical trial, part of a multicenter study carried out in three centers, two in Brazil and one in the United States. This study followed the CONSORT (Consolidated Standards of Reporting Trials) guidelines for the submission of clinical trials and was registered in the Brazilian Registry of Clinical Trials (*Registro Brasileiro de Ensaios Clínicos*, REBEC) under registration code RBR-4bhr78.

### Locus

The study was carried out at two public universities in the state of Paraná. The first university is located in the state’s capital, with more than 45 years since the creation of the Nursing course; it has 10 periods for completion and admission of new students occurs via the entrance exam/National High School Exam (*Exame Nacional do Ensino Médio*, ENEM); it has a mean of 30 new vacancies every six months. The second institution is located in the inland of the state (approximately 200 km from the Capital), with a mean annual admission of 40 students via entrance exam/ENEM. In both universities, critical thinking and clinical reasoning are addressed throughout the course by means of case studies, simulation, practical classes and active methodologies in theoretical classes. The clinical practices are intensified in the second half of the course.

### Period

Data collection took place between February and June 2019, on different days in each class at each university.

### Population

The research participants were students attending four classes of the Undergraduate Nursing Courses. The evaluators of the resolution of the case studies also participated in the research.

### Selection criteria

The following inclusion criteria were listed: being a student regularly enrolled in the undergraduate Nursing course, not having a medical diagnosis of dyslexia and being present in the first stage of data collection; having attended, with passing grades, the academic disciplines of Nursing Care Fundamentals and Nursing in Adults’ and Older Adults’ Health or Practical Foundations for Nursing Care and Adults’ and Older Adults’ Health. These academic disciplines were chosen because the NP approach takes place in them, with teaching of the NANDA-I ND taxonomy in the research institutions. The research exclusion criterion was not solving the case study in the second phase. 

Two case study resolution evaluators were selected from the multicenter study team, based on the working locus. All team members are nurses, experienced in the NP. In case of doubts, a third researcher from the team was consulted for evaluation, due to the experience in correcting resolutions in another participating center.

### Sample definition

**Figure 1:**
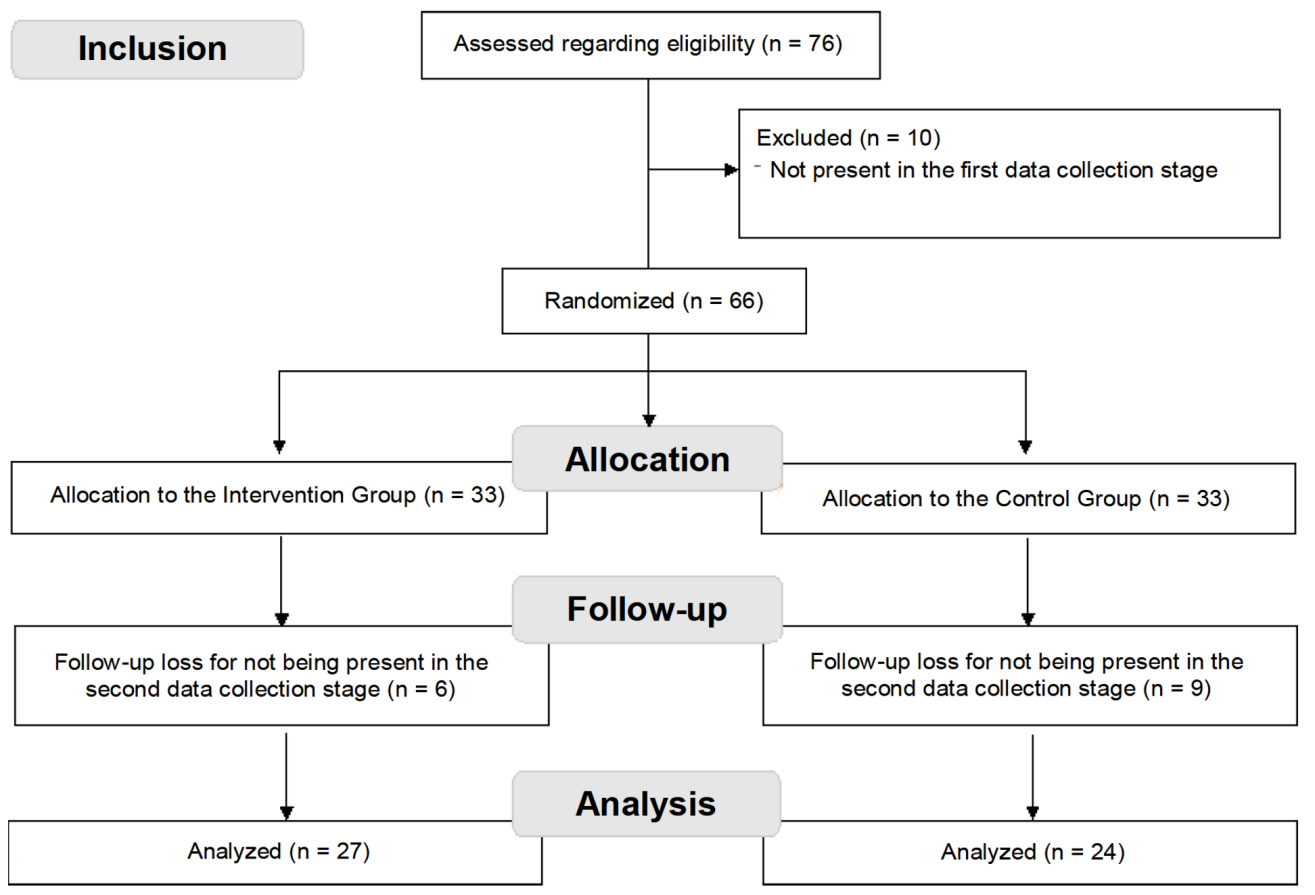
Data collection flowchart according to CONSORT

The sample was determined by convenience, according to the students’ availability for data collection at each study locus. At the time the data for the first phase were collected, there were 76 students enrolled in the academic disciplines in question at both universities. In the first phase, there was a total of 66 students present, who agreed to participate, and in the second phase there were 51; the 15 absences were excluded from the study; leaving 51 students, 27 allocated to the intervention group and 24 to the control group, according to the random sequence shown in Figure 1.

### Variables

The independent variables investigated include: demographic ones (gender, age), academic ones [schooling (whether first bachelor’s or previous bachelor’s degree in another field), the student’s perception of confidence in identifying the patient’s NDs/problems, the student’s perception regarding the knowledge needed to identify the patient’s NDs/problems and causes, student’s perception regarding confidence in the identification of important clues to identify the patient’s priority NDs, and the student’s perception regarding the use of knowledge from the care provided to a similar patient or personal experience in the analysis of the case].

The outcome variables were as follows: accuracy in identifying the patient’s ND/problem, accuracy in identifying the appropriate etiologies for the patient’s ND/problem, and number of relevant clues (signs and symptoms or defining characteristics) that confirm the patient’s priority ND. 

### Instruments used

A demographic questionnaire was used to measure the gender, age and schooling variables. To assess the student’s perception regarding confidence, knowledge and use of the knowledge, a questionnaire on decision-making was used with questions such as “How confident do you feel in identifying the patient’s NDs/problems?” and “Do you think you had the necessary knowledge to identify the patient’s priority NDs/problems and causes in both case studies?”. These materials were created by the American researcher, author of the Guide, and also translated into Portuguese.

To verify accuracy in identifying the patient’s ND/problem and etiologies, a scoring rubric created by the author of the instrument was used. This item was created based on the Lunney Scoring Method, a semantic differential scale to estimate the accuracy of Nursing diagnoses which assigns values from -1 to 5 to the NDs/problems identified in the patient^15^. The rubric for the accuracy score was previously defined by experts in patients’ NDs/problems, taking into account the case studies analyzed. 

The version used in this research, both of WCRP and rubrics, case studies and other instruments, is the one translated and adapted to Brazilian Portuguese^14^.

### Data collection

Through randomization of the control and intervention groups, it was sought to know the effects of WCRP on diagnostic and etiological accuracy, as well as on the identification of validated case study clues, with evaluation through a rubric. The activity required the student to identify an ND or priority problem of the patient, the etiology (cause) of the ND and the clues (signs and symptoms) that justify it, without consulting any materials (Internet, books, notes and others). A baseline evaluation (Phase 1) was performed, followed by a second evaluation (Phase 2), after randomization and allocation of the students in the Intervention and Control groups, with the Intervention Group using WCRP in the second evaluation. In order to minimize the possibility of sample loss, the researchers and professors responsible for the academic disciplines encouraged participation in both phases. However, to avoid contamination of the sample submitted to the intervention, Phase 2 was carried out in a single day, previously agreed upon with the students.

The priority ND for both cases is “Ineffective airway clearance” from NANDA-I, or a problem that was compatible with the ND description related to partially obstructed airway, airway spasm, excessive mucus and/or retained secretions. Both cases include eight identical clues (signs and symptoms) that justify the priority ND^9^. 

At both moments, the evaluations of the intervention and control groups using the rubric were carried out in consensus by two researchers who are members of the multicenter study group and, in case of doubts, a third researcher was consulted to reach consensus. Answers left blank, or marked as “I don’t know”, were excluded from the analysis. Descriptive statistics were used to measure accuracy of the clues, with absolute and relative frequencies of those correctly identified, according to the NANDA-I Nursing Diagnosis Classification^9^.

The Nursing students were invited to participate in the study during undergraduate class hours, according to the availability and prior authorization of the students of the corresponding academic discipline. It was advised that the students’ participation was voluntary and independent of the evaluation in the course.

The envelope delivery sequence (intervention and control) containing the instructions, the case study and instruments in the second phase was determined by means of randomization to the groups, according to the sequence previously generated on the random.org website. The researchers who worked in data collection, as well as the rubric evaluators, were oblivious to the allocation of students in the intervention or control groups. 

### Data treatment and analysis

The data were analyzed using the R software, version 3.5.1, and in Microsoft Excel, with the help of a statistician. Descriptive statistics were used to present the demographic and academic data, by treatment group (intervention and control) and moment (baseline and second evaluation). Fisher’s Exact test was used to compare the two groups in terms of prior schooling, gender and perceptions of confidence, knowledge, and knowledge use. To compare the mean ages between the groups, Mann-Whitney’s test was used. The non-parametric ANOVA test for repeated measures was used to test the effect of time on the outcomes (comparing the students’ intragroup scores between Phase 1 and Phase 2); to test the group effect (comparing the students’ scores between Phase 1 and Phase 2); and to test the effect of the interaction between time and group factors (comparing the score evolution over time in the intervention group with the score evolution over time in the control group). When there was an interaction effect, Mann-Whitney’s Paired Test was performed within each group. Type I error was set at 5% as statistically significant. 

### Ethical aspects

The multicenter project was authorized by the Institutions and submitted to the Research Ethics Committees of both Universities, obtaining due approval. Inclusion of the participants followed the recommendations for research studies involving the participation of human beings, according to Resolution 466/12 of the National Health Council. All participants signed the Free and Informed Consent Form (FICF). All were instructed regarding the study objectives and the possibility of withdrawing at any phase of the research.

## Results

A total of 51 students participated in the research, 24 allocated to the control group and 27 to the intervention group. There were no differences between the groups in relation to the demographic and academic characteristics (Table 1).

**Table 1:** Demographic and academic characteristics of the students. Curitiba, PR, Brazil, 2020

Characteristics	Intervention Group - (n=27)	Control Group (n=24)	Total	p-value
	n (%)	n (%)	n (%)	
**Age (years old), mean±standard deviation**	22.2±3.2	21.8±2.0	22±2.7	0.950*
**Gender**				
Female	22 (81.5)	20 (83.3)	42 (82.4)	1.00^†^
Male	5 (18.5)	4 (16.7)	9 (17.6)	
**Schooling**				
First graduation	27 (100.0)	23 (95.8)	50 (98.0)	0.476^†^
Bachelor’s degree in another field	0 (0.0)	1 (4.2)	1 (2.0)	
**Perceived confidence to identify diagnoses**				
Not at all confident	1 (3.7)	3 (12.5)	4 (7.8)	0.234^†^
Somewhat confident	12 (44.4)	13 (54.2)	25 (49.0)	
Confident	14 (51.9)	7 (29.2)	21 (41.2)	
No answer	0 (0.0)	1 (4.2)	1 (2.0)	
**Perceived confidence in the knowledge needed to identify diagnoses and causes**				
A little	5 (18.5)	8 (33.3)	13 (25.5)	0.332^†^
Very much	21 (77.8)	15 (62.5)	36 (70.6)	
Total	1 (3.7)	0 (0.0)	1 (2.0)	
No answer	0 (0.0)	1 (4.2)	1 (2.0)	
**Perceived confidence in the knowledge needed to identify important clues**				
Not at all confident	0 (0.0)	1 (4.2)	1 (2.0)	0.488^†^
Somewhat confident	8 (29.6)	10 (41.7)	18 (35.3)	
Confident	17 (63.0)	11 (45.8)	28 (54.9)	
Very confident	2 (7.4)	1 (4.2)	3 (5.9)	
No answer	0 (0.0)	1 (4.2)	1 (2.0)	
**Perception regarding the use of knowledge from the care provided to a similar patient/personal experience**				
Yes	11 (40.7)	10 (41.7)	21 (41.2)	
No	16 (59.3)	13 (54.2)	28 (54.9)	
No answer	0 (0.0)	1 (4.2)	1 (2.0)	

^*^Mann-Whitney’s test; ^†^Fisher’s Exact test

There was a significant effect of the time factor (T) on diagnostic accuracy (p=0.041), indicating that there is an intragroup difference regarding diagnostic accuracy between Phase 1 and Phase 2, although there was no intergroup difference. The mean diagnostic accuracy was higher in Phase 1 than in Phase 2.

It is observed that there was no significant effect of the group (G), Phases 1 and 2 (T) and the interaction (I) between Group and Time for the clues. This indicates that the number of clues identified in Phases 1 and 2 did not change in both groups (Table 2).

**Table 2:** Diagnostic accuracy, etiological accuracy, clues and response time according to group and application moment. Curitiba, PR, Brazil, 2020

Phases/Groups	Intervention Group - (n=27)					Control Group - (n=24)					p-value^‡‡^
	M^*^	SD^†^	SD^‡^	Q1^§^	Q3^||^	M^*^	SD^†^	SD^‡^	Q1^§^	Q3^||^	
**Diagnostic accuracy**											G^¶^:0.390; T^**^:0.041; I^††^:0.270
**Phase 1**	2.04	2.34	3.00	-1.00	4.00	3.09	1.73	4.00	3.00	4.00	
**Phase 2**	1.93	2.13	3.00	-1.00	4.00	1.67	2.57	3.00	-1.00	4.00	
**Etiological accuracy**											G: 0.905; T: 0.122; I: 0.003
**Phase 1**	3.59	1.19	3.00	3.00	5.00	3.21	0.59	3.00	3.00	3.00	
**Phase 2**	3.04	0.65	3.00	3.00	3.00	3.38	0.71	3.00	3.00	3.25	
**Clues**											G: 0.211; T: 0.896; I: 0.678
**Phase 1**	4.22	1.42	4.00	3.00	5.00	4.75	1.48	4.50	4.00	6.00	
**Phase 2**	4.15	1.85	5.00	3.00	5.00	4.62	1.56	4.00	3.75	6.00	

^*^M = Mean; ^†^SD = Standard Deviation; ^‡^Md = Median; ^§^Q1 = First quartile; ^||^Q3 = Third quartile; ^¶^G = Groups; ^**^T = Time; ^††^I = Interaction; ^‡‡^Non-parametric ANOVA test for repeated measures

Regarding etiological accuracy, there was an interaction effect between Group and Time, indicating that the evolution of the groups occurred in a differentiated manner. Thus, after analysis within each group using Mann-Whitney’s paired test, there was a significant change between Phases 1 and 2 in the intervention group (p=0.0351), but not in the control group (p=0.5385). 

Table 3 represents the absolute and percentage frequencies of the scores for both groups, at the two moments, for diagnostic accuracy and etiological accuracy. In relation to diagnostic accuracy, the intervention group more frequently indicated diagnoses with a score of +4, both in Phase 1 (n=11; 40.7%) and in Phase 2 (n=9; 33.3%). In the control group, this occurred in Phase 1 (n=10; 43.5%) but, in Phase 2, the most frequent score was -1 (n=11; 45.8%). Regarding etiological accuracy, the score +3 was the most frequent, at both moments and in both groups.

**Table 3:** Score referring to diagnostic accuracy and etiological accuracy. Curitiba, PR, Brazil, 2020

Groups/Rubric score	Intervention Group - (n=27)		Control Group - (n=24*)	
	Phase 1 - n (%)	Phase 2 - n (%)	Phase 1 - n (%)	Phase 2 - n (%)
Diagnostic accuracy				
+5	1 (3.7)	0 (0.0)	2 (8.7)	3 (12.5)
+4	11 (40.7)	9 (33.3)	10 (43.5)	6 (25.0)
+3	5 (18.5)	6 (22.2)	8 (34.8)	4 (16.7)
+2	0 (0.0)	3 (11.1)	0 (0.0)	0 (0.0)
+1	0 (0.0)	0 (0.0)	0 (0.0)	0 (0.0)
0	1 (3.7)	1 (3.7)	0 (0.0)	0 (0.0)
-1	9 (33.3)	8 (29.6)	3 (13.0)	11 (45.8)
Etiological accuracy				
+5	9 (33.3)	2 (7.4)	2 (8.3)	3 (12.5)
+4	1 (3.7)	0 (0.0)	1 (4.2)	3 (12.5)
+3	16 (59.3)	22 (81.5)	21 (87.5)	18 (75.0)
+2	0 (0.0)	3 (11.1)	0 (0.0)	0 (0.0)
+1	0 (0.0)	0 (0.0)	0 (0.0)	0 (0.0)
0	1 (3.7)	0 (0.0)	0 (0.0)	0 (0.0)
-1	0 (0.0)	0 (0.0)	0 (0.0)	0 (0.0)

*In relation to diagnostic accuracy, one participant in the Control Group answered “I don’t know”. The answer was not counted, according to the method. Therefore, n=23 was considered for diagnostic accuracy in the control group

Regarding the eight clues related to the priority ND contained in the case studies (snoring, wheezing, tachypnea, difficulty verbalizing, dyspnea, coughing, inability to clear secretions and orthopnea), a higher frequency of identification of tachypnea and difficulty verbalizing (n=19; 70.4% in both) was detected; and the wheezing (n=20; 74.1%) and dyspnea (n=19; 70.4%) clues in the second evaluation. In the control group, in the baseline evaluation, the dyspnea (n=19; 79.2%), coughing and wheezing (n=17; 70.8) clues were more frequently observed; while snoring and wheezing (n=20; 83.3%) were identified in the second evaluation. Orthopnea was the least identified clue in both groups, results shown in Table 4.

**Table 4:** Absolute and percentage frequencies of the diagnostic clues identified. Curitiba, PR, Brazil, 2020

Clues	Intervention Group - (n=27)		Control Group - (n=24)	
	Phase 1 - n (%)	Phase 2 - n (%)	Phase 1 - n (%)	Phase 2 - n (%)
Snoring	15 (55.5)	13 (48.1)	16 (66.7)	20 (83.3)
Wheezing	15 (55.5)	20 (74.1)	17 (70.8)	20 (83.3)
Tachypnea	19 (70.4)	16 (59.3)	13 (54.2)	14 (58.3)
Difficulty verbalizing	19 (70.4)	12 (44.4)	15 (62.5)	16 (66.7)
Dyspnea	13 (48.1)	19 (70.4)	19 (79.2)	16 (66.7)
Coughing	16 (59.3)	17 (63.0)	17 (70.8)	14 (58.3)
Inability to clear secretions	17 (63.0)	15 (55.5)	15 (62.5)	10 (41.7)
Orthopnea	0 (0.0)	0 (0.0)	2 (8.3)	1 (4.2)

## Discussion

There was predominance of female students, with a mean age of 22 years old and attending their first undergraduate course, results that are in line with other studies conducted with Nursing students in Brazil and in the world^16^ ^-^ ^18^. As for the results referring to diagnostic accuracy, it was observed that the mean, both in the control group and in the intervention group, decreased from Phase 1 to Phase 2, with statistical significance. This result contradicts the initial hypothesis that WCRP would increase the students’ diagnostic accuracy. 

In a recent research study carried out with Nursing students at a university in Alabama-United States, one of the objectives was to measure the ability of clinical reasoning before and after implementing the OPT (Outcome Present State Test) model during the students’ clinical experiences, using the Health Sciences Reasoning Test to compare the results. OPT aims at providing a framework for nurses/students to assess and analyze the patient’s data and identify the current clinical problem and desired outcome. The test results showed that the students had better scores in the first phase, with a significant difference (p=0.018). The author pointed out the accumulation of activities and tests at the end of the semester, and the fact that the model was introduced in that semester, without any face-to-face explanation, as important factors that may have influenced the students’ performance^19^. In this study, data collection was carried out predominantly at the beginning of the semester or at the end of all activities, possibly without interference from the stress inherent to the activities. However, in the study locus, it was shown that one-time self-instruction was not enough to exert an impact on the students’ diagnostic accuracy. 

A quasi-experimental study carried out in Indonesia examined the impact of a teaching strategy that used cognitive learning principles on accuracy, inaccuracy and self-confidence in nursing students’ clinical reasoning. The results showed that the six-week educational intervention had significant positive effects on the development of clinical reasoning skills, with increased accuracy in Phase 2 in the intervention group (p<0.00). The authors argue that the interaction of the discussion of specific case scenarios, in small groups, and the teacher-led learning experience, can help to achieve successful results from the strategy^20^.

In this way, Nursing education is understood as something procedural, continuous and that outlines the professional profile of those undergoing training, and it is here that the fundamental role of the professor as a facilitator of this process stands out^21^. A research study carried out to assess applicability of the DNT model in Brazil - the model that supported the creation of WCRP - pointed out the students’ experience as positive for the development of diagnostic accuracy. However, even though it is considered as a tool in which the student participates actively, it was suggested that the DNT model be applied with prior explanation from the professor, with repeated applications in other contexts^22^. This study was carried out concurrently with the creation and testing of the American WCRP. Thus, the strategy of repeated applications cannot be implemented in the current study of the implementation of WCRP in Brazil, as it was a multicenter study, already initiated in the USA center.

Analyzing the students’ score for diagnostic accuracy, according to the Lunney Scoring Method, it is noticed that the most accurate priority ND was discreetly mentioned by the students at both moments and in both groups, being more frequent in the control group. This is the “Ineffective airway clearance” ND, defined as “Inability to clear secretions or obstructions from the respiratory tract to maintain a clear airway”^9^. This clinical condition requires immediate action due to the risk of death or sequelae caused by the reduction of circulating oxygen, especially in children and older adults, who are part of the commonly affected population, also represented in the research case studies. Its accurate identification allows for a rapid intervention and can minimize the hypoventilation effects^23^. 

On the other hand, the second most accurate ND, “Ineffective breathing pattern”, was the most frequent in the answers given by the students in both groups, in Phases 1 and 2. It is defined as “Inspiration and/or expiration that does not provide adequate ventilation”^9^. “Impaired gas exchange” was the third ND most frequently mentioned by the students as a priority, but this corresponds to the least accurate (-1), according to the scoring rubric, for both cases. This is defined as “Excess or deficit in oxygenation and/or in the elimination of carbon dioxide at the alveolar-capillary membrane”^9^. 

Nursing diagnoses related to respiratory problems have some defining characteristics in common, generating doubts for the less experienced, such as the students. It is known that identifying inaccurate NDs can compromise patient care, leading to an inadequate care plan and, consequently, to inappropriate results for the clinical situation of the evaluated individuals^7^ ^,^ ^9^, exposing them to risk. 

Despite having similar defining characteristics, such as dyspnea, orthopnea, restlessness and others, these NDs have different central conceptions, which can be differentiated by the professional who masters concepts such as ventilation, airway patency and pulmonary gas exchange. Therefore, although each of these diagnoses is related to the respiratory system, they have a divergent central concept. The understanding of central concepts, such as ventilation, gas exchange, breathing pattern and permeability, is necessary so that the professional does not omit important data and recognizes normal and abnormal patterns^9^.

As for the results obtained regarding the etiology of the main diagnosis, the analysis also indicated a different result than the expected. The intervention group had a worse performance when compared to the control group after using the Guide, in opposition to the initial study hypothesis. A statistically significant difference was observed between the mean values of Phases 1 and 2 in the intervention group, which represented a lower mean in Phase 2.

In the taxonomy used, etiologies are referred to as related factors that include circumstances, facts or influences that have a certain type of relationship with the ND (e.g., cause, contributing factor). Correctly evaluating the etiological factors that determine the health problems is part of the diagnostic process performed by nurses^6^. Adequate identification is necessary so that, whenever possible, the interventions are aimed at these etiological factors, seeking to remove the underlying cause of the ND^9^. 

A research study carried out with Nursing students (n=50) sought to identify the phases of the Nursing process in which the students found greater difficulties, through the application of a validated case study. It was found that more than half of the students misidentified the factors related to the diagnoses indicated, and performed better in identifying the defining characteristics, expected results and interventions^24^. Another study analyzed 897 Nursing students’ care plans and found that 45.8% of them did not achieve proficiency in identifying the etiological factors of the diagnoses in question^25^.

It is crucial to understand how Nursing students use the related factors to identify the NDs, as diagnostic accuracy is based on the ability to connect these factors to better represent the patient’s current condition^26^.

In the current study, the students identified, more frequently, the causes with a score of +3 on the diagnostic accuracy scale, which, according to the rubric of the case studies, are as follows: hyperplasia of the bronchial walls, history of exposure to smoke/poor quality air/smoker spouse and COPD (Chronic Obstructive Pulmonary Disease) history. These factors are present in the case studies, but they do not represent the main etiology of the priority problem, which, by the rubric, could be described as: partially obstructed airway, airway spasms, excess mucus and retained secretions.

The importance of knowing the factors related to the NDs to obtain clinical evidence when planning and implementing the care plan is highlighted. There is lack of scientific production on this theme, with a gap in studies that address the accuracy of the factors related to the respiratory NDs and teaching in the training of nurses^27^.

When the results referring to the clues identified were analyzed, decreasing mean values were obtained in both groups, although without statistical significance. Clues, defining characteristics or signs and symptoms are obtained during the interview and physical examination of the patient, and correspond to the grouped manifestations of the ND^9^. The importance of the information obtained at that moment is emphasized, which will support decisions regarding the diagnoses, Nursing interventions and evaluation of the results^6^. The ND accuracy validation occurs when the nurse identifies and correctly associates the defining characteristics with the related factors according to the patient’s evaluation. Identifying the defining characteristics supports ND accuracy^9^.

A thoughtful analysis of the signs and symptoms presented by the patients, as well as an understanding of their relevance, support adequate decision-making. For example, if the students of this research paid attention to the fact that O_2_ saturation be adequate, many would possibly not have pointed out “Ineffective spontaneous ventilation” or “Improved gas exchange” as a priority diagnosis, as the excessive presence of mucus was crucial for the diagnosis of ineffective airway clearance. Thus, NDs that are not well-supported, through defining characteristics, related or risk factors, are not appropriate for a patient^9^.

It is interesting to note that orthopnea is identified as a good clinical indicator of “Ineffective airway clearance”; however, in the current study it was a clue scarcely mentioned by the students, despite also being present in respiratory NDs, namely: “Impaired gas exchange” and “Ineffective breathing pattern”^9^ ^,^ ^27^. Orthopnea corresponds to the sensation of dyspnea in the horizontal position; it is relieved, totally or partially, with elevation of the headboard or use of more pillows; and can be seen in patients with COPD. The supine position causes elevation of the abdominal viscera, generating opposition to the diaphragmatic inspiratory incursions, a complicating factor in this population^28^. Observation of this symptom provides evidence for the nurse to prescribe elevation of the headboard and provide comfort to the patient^29^.

Some limitations of this research should be pointed out. It was not possible to collect the first and second phases with the same expected time interval (two weeks) due to the long holiday (carnival) and to the fact that the dates given depend on the availability of the professors in charge of the academic disciplines to release students for the activity. The reduced sample size and the non-uniformity of days and times for data collection may have also influenced the results obtained. It is suggested that, if possible, these factors are controlled in a future application, reducing a possible bias. Despite the invitation and encouragement by the professors in charge of the Academic Disciplines, there was a significant follow-up loss from Phase 1 to Phase 2 of the research (22.7%). A strategy to minimize the follow-up loss would be to carry out the second phase on different days, in order to rescue the missing participants. This strategy, however, could lead to sample contamination, through conversations with students who have already solved the clinical case.

The results of this study contribute to the future choice of interventions to improve the Nursing students’ diagnostic clinical reasoning. In settings where self-instruction is interesting, as in the case of a large number of students, it is inferred that application of the guide should be repeated, continuous and integrated with the contents, throughout the semester, and using active teaching methodologies. Furthermore, it is desirable to reinforce the teaching of academic disciplines in the basic cycle, such as Anatomy, Physiology and Pathology, among others, enabling integration with the Nursing practice, in order to explain nurses’ thinking and reasoning in different situations.

## Conclusion

All the hypotheses established in this research were rejected, that is, that WCRP would improve accuracy in identifying the patient’s priority ND, etiology and signs and symptoms. 

It is recommended that other studies be carried out with the purpose of evaluating effectiveness of the guide, controlling the limitations pointed out and following the recommendations. The most relevant of them refers to continuous use, applying the pillar of repetition, as presupposed by the Theory that underlies the instrument. In this way, WCRP may be tested in a different way from the one applied in this research and, perhaps, contribute to the training of Nursing professionals that add quality to care by precisely identifying the priority NDs, generating appropriate interventions, and contributing to patient safety.
